# Data-driven microstructural optimization of Ag-Bi-I perovskite-inspired materials

**DOI:** 10.1038/s41524-025-01701-7

**Published:** 2025-07-03

**Authors:** Kshithij Mysore Nandishwara, Shuan Cheng, Pengjun Liu, Huimin Zhu, Xiaoyu Guo, Fabien C.-P. Massabuau, Robert L. Z. Hoye, Shijing Sun

**Affiliations:** 1 Department of Mechanical Engineering, University of Washington, Seattle, WA USA; 2 Inorganic Chemistry Laboratory, Department of Chemistry, University of Oxford, South Parks Road, Oxford, UK; 3 Department of Physics, SUPA, University of Strathclyde, Glasgow, UK

**Keywords:** Materials for devices, Theory and computation

## Abstract

Microstructural design is crucial yet challenging for thin-film semiconductors, creating barriers for new materials to achieve practical applications in photovoltaics and optoelectronics. We present the Daisy Visual Intelligence Framework (Daisy), which combines multiple AI models to learn from historical microscopic images and propose new synthesis conditions towards desirable microstructures. Daisy consists of an image interpreter to extract grain and defect statistics, and a reinforcement-learning-driven synthesis planner to optimize thin-film morphology. Using Ag-Bi-I perovskite-inspired materials as a case study, Daisy achieved over 120× and 87× acceleration in image analysis and synthesis planning, respectively, compared to manual methods. Processing parameters for AgBiI_4_ were optimized from over 1700 possible synthesis conditions within 3.5 min, yielding experimentally validated films with no visible pinholes and average grain sizes 14.5% larger than the historical mean. Our work advances computational frameworks for self-driving labs and shedding light on AI-accelerated microstructure development for emerging thin-film materials.

## Introduction

Thin-film semiconductors are key enablers of next-generation photovoltaics, optoelectronics, microelectronics, and quantum information technologies^1–5^. Over the past decade, lead-halide perovskite semiconductors have attracted significant attention for their high solar-to-electricity power conversion efficiencies, lightweight properties, and compatibility with low-temperature, cost-effective fabrication processes^6–9^. More recently, driven by increasing sustainability needs, significant efforts have been focused on developing new materials to address degradation challenges by replacing volatile organic species in alkylammonium metal halides with inorganic counterparts, such as Cs, Rb and Ag^10^, as well as mitigating toxicity by substituting Pb with Sn, Bi, Sb, or Cu^11^. However, achieving high-quality thin films using lead-free perovskite-inspired materials remains challenging, creating a significant gap between materials discovery and realization of their device performance potential. A critical missing link here is microstructural design, where morphological features such as grain size and defect coverage of thin-film semiconductors dictate their optoelectronic properties, ranging from charge-carrier mobility, recombination rates, to overall device efficiency^12^. As an example, we can consider Ag-Bi-I compounds (e.g., AgBiI_4_, Ag_2_BiI_5_ or Ag_3_BiI_6_), which have gained interest for indoor photovoltaics applications because of their high optical limits in efficiencies exceeding 50% white light emitting diode illumination^13–16^. As with other thin film materials, improved charge-carrier transport, and improved photovoltaic performance are achieved with larger grains, continuous film morphology, and lower defect densities^17,18^. However, this is challenging to achieve, and the performance of Ag-Bi-I-based devices have fallen well below their theoretical limits^14^. A new approach to accelerate the development of defect-free, uniform, and large-grained thin films is therefore crucial for shortening the lab-to-market timeline transitioning new semiconducting materials to practical applications^19^.

Microstructural design is inherently complex and labor intensive, requiring advanced characterization to reveal film morphology and deep understanding of the process-microstructure relationships across large synthetic spaces. For emerging lead-free perovskite-inspired materials, state-of-the-art solution-processed thin film optimization workflows typically involves applying precursor/substrate preheating (i.e., hot casting), spin coating speed profile tuning, or antisolvent addition during synthesis, followed by examining the film morphology, for example with scanning electron microscopy (SEM)^20^. Taking characterization feedback to develop optimization strategies for subsequent synthesis remains a significant rate-limiting step, hindering the development of emerging lead-free perovskite-inspired materials, and the exploration of new compositions. Traditionally, extracting scientific insights from microscopy images is performed by materials scientists, relying on expert judgment, sample-by-sample, to identify defective features and grain statistics. However, manual interpretation is inherently slow, non-scalable, and prone to human bias. To date, some progress has been made through computer vision using machine learning (ML) models to automate image analysis^21^. For instance, Chen et al. implemented a logistic regression-based classifier for edge detection in SEM images of perovskite films, outperforming traditional non-ML methods like Canny Edge Detections^22^. Zhang et al. developed a U-Net-based convolutional neural network (CNN) to quantify individual grain surface areas in perovskite films, using a Voronoi-inspired post-processing method to improve accuracy and uncover statistically reliable grain descriptors linked to device performance^23^. Beyond perovskite semiconductors, Barakati et al. introduced a physics-based reward-driven image analysis framework, leveraging Bayesian optimization and reward functions to optimize microscopy workflows dynamically^24^. This approach enables real-time optimization of complex analysis tasks, offering improved precision and efficiency compared to deep CNNs, particularly in noisy imaging conditions. Existing data-driven image analysis primarily focuses on post-synthesis analysis based on pre-labeled image databases, nevertheless, there is a lack of mechanisms to translate characterization results into actionable guidance for adjusting synthesis parameters in response to observed microstructures. Several studies to date have reported predictive modeling that takes synthetic parameters as input to generate the expected microstructural outcome^25–27^. However, a significant gap exists in the intelligent generation of synthetic plans based on automated learning from visual information at a microscale, which is a an essential capability for realizing the full potential of self-driving laboratories and achieving full automation.

In this work, we present a new visual intelligence framework, namely the Daisy Framework (Daisy), which integrates advanced computer vision techniques with an optimization agent to build the feedback loop between characterization and next round synthesis, effectively moving towards a self-driving lab paradigm for accelerated thin-film microstructure optimization. The Daisy framework was trained on the existing experimental data collected entirely by the authors over several months, which includes synthesis and SEM characterization of AgBiI_4_, Ag_2_BiI_5_, and Ag_3_BiI_6_, demonstrating direct learning of an AI agent from historical laboratory data (see Fig. S1 for workflow overview). The framework is designed to contain two sub-systems: a goal-oriented image interpreter and a one-shot synthesis planner. Being trained on 161 SEM images of Ag-Bi-I thin films synthesized in-house, the Daisy image interpreter consists of three interconnected AI models that perform segmentation, classification and clustering tasks, respectively, allowing automated quantification of grain characteristics and defect coverage without manual labeling and a 97% accuracy was achieved. The Daisy synthesis planner consists of an offline reinforcement learning (RL) agent that takes the results from the image interpreter and searches for optimal processing parameters towards defect-free, large grain thin films. Our case study applying the Daisy synthesis planner to AgBiI_4_ was experimentally validated, where the AI agent identified an unexplored region in the processing parameter space showing desirable microstructural features. This discovery led to an AgBiI_4_ film with a 0% visible defect percentage and a 14.5% increase in grain size compared to the mean grain size of all defect-free samples within the historical dataset. Our approach is broadly applicable beyond Ag-Bi-I semiconductors, offering a key advancement in AI-driven materials optimization. By leveraging visual data for autonomous decision-making, it represents a transformative step toward fully automated microstructural design for early-stage thin-film semiconductors.

## Results

### Daisy image interpreter

**Fig. 1:**
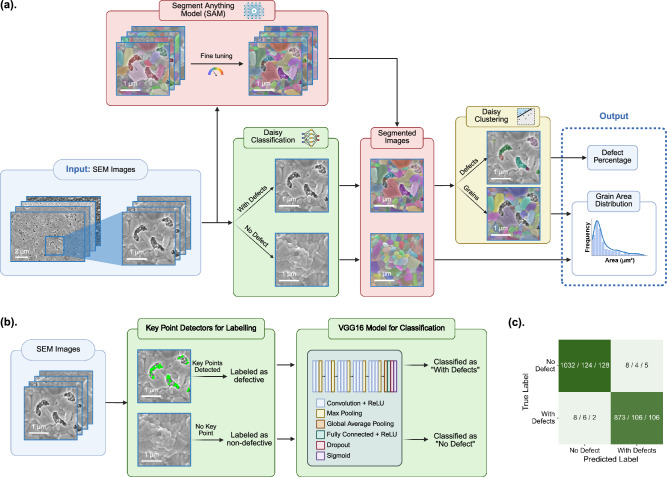
Workflow of the Daisy Image Interpreter. **a** Workflow showing the integration of Daisy segmentation, classification, clustering models, and the data extraction process of grains and defects from scanning electron microscopy (SEM) images. Blue blocks indicate the model’s input and output, red blocks represent the Segment Anything Model (SAM) for segmentation, green block denotes the classification algorithm, and yellow block signifies the clustering algorithm. **b** Workflow illustrating the model architecture in the Daisy classification algorithm. **c** Classification confusion matrix for the Daisy classification model. Each entry in the confusion matrix is represented in the format of A / B/ C, where A is the number of image patches on the training set (80% of data), B is on the validation set (10% of data), and C is on the test set (10% of data).

Typical SEM images of Ag-Bi-I thin films show densely packed sub-micron-scale grains separated by irregularly circular grain boundaries, punctuated by regions devoid of the semiconductor, called “pinholes”. These pinholes cause shunting and severe reductions in photovoltaic device performance, and it is critical to minimize or eliminate them. The primary objective of the Daisy Image Interpreter is to extract grain-specific characteristics from each SEM image. To avoid the tedious manual labeling process for model training, we developed three interconnected models, namely, Daisy segmentation, classification, and clustering, respectively, to collectively automate the information extraction processes. Following initial data preprocessing steps (as detailed in Methods), we first fine-tuned the Segment Anything Model (SAM) for image segmentation, dividing each image into hundreds of grain regions for detailed analysis^28^. SAM generates segmentation masks by identifying areas with distinct visual characteristics, such as texture and intensity. To enhance segmentation accuracy, we refined SAM through iterative adjustments, specifically by tuning parameters like points per side and Intersection-over-Union (IoU) thresholds, which allowed us to capture finer structural details and increase mask stability^29,30^. We also applied dynamic filtering to remove segments exceeding 10% of the total image area, focusing attention on microstructural features at the length scale of single grains. The outcome of our model refinements is illustrated inside the red block in Fig. 1a, where the segmented image displays a significantly larger number of detected regions, represented in different colors, compared to the initial segmentation output without fine tuning.

**Fig. 2:**
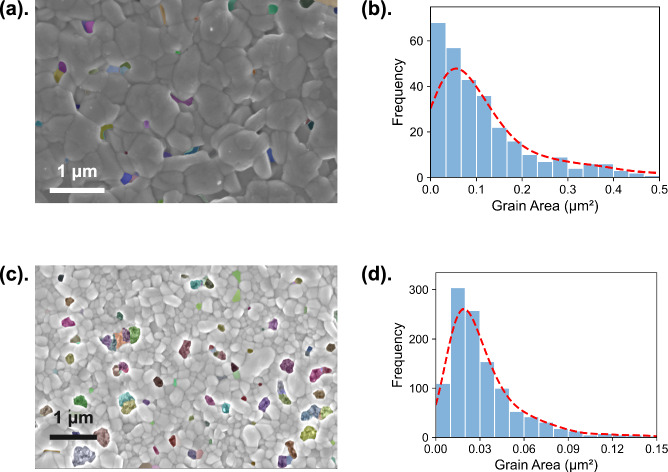
Representative results from the Daisy Image Interpreter. **a** Scanning electron microscopy (SEM) image of an AgBiI_4_ thin film, showing segmented defects highlighted in color, covering 5.2% of the total area. The image, captured at a magnification of 51,390×, was processed by the Daisy Image Interpreter in 29 s. **b** Grain area distribution corresponding to the thin film in (**a**), with the red dashed line indicating the kernel density estimation of the distribution. **c** SEM image of another AgBiI_4_ thin film, showing segmented defects highlighted in color, covering 18.3% of the total area. The image, captured at a magnification of 45,360×, was processed by the Daisy Image Interpreter in 38 s. **d** Grain area distribution corresponding to the thin film in (**c**), with the red dashed line indicating the kernel density estimation of the distribution.

The results from image segmentation show that grain-level information has been successfully extracted from SEM images; however, information at this stage remains incomplete. We found that many SEM images contain defects, i.e., pinholes, as illustrated in Fig. 2a, c. Without domain-specific labeling, segmentation algorithms identify grains and defects as individual features without distinguishing between them. In other words, microscopic features were isolated but not recognized in the materials science context. To address this, we developed a data-driven approach consisting of two steps: first, a classification model was trained to distinguish between SEM images with and without defects. Images identified as defect-free proceed directly to grain analysis without the additional clustering step, thereby saving computational time. For images identified as containing defects, a subsequent clustering algorithm was applied to the segmented images, which enables differentiation between defective features such as pinholes and irregular grains in each SEM image. Our clustering method was able to effectively distinguish grains from defects and here we employed unsupervised ML to avoid the need for pre-training labeling, which both saved significant data preparation time and allowed the model transferability to apply the same approach to new, unlabeled datasets readily. As shown in the yellow block in Fig. 1a, the Daisy clustering algorithm was applied to all images classified as “with defects”. Using feature maps generated by the fine-tuned SAM, the clustering algorithm employed k-means clustering to iteratively partition pixels into clusters, each corresponding to distinct microstructural elements. By refining clusters according to size thresholds and filtering out noise-related clusters as detailed in the Methods section, we achieved effective separation of grains and defect areas.

Prior to clustering, the distinction between SEM images with and without defects was achieved by a separate Daisy classification model. Unlike segmentation and clustering algorithms that were applied to all pixels in a SEM image, the classification model generates a single label of “defective” and “no defect” for each SEM image. To construct the training dataset for classification, 141 images were selected from the 161 historical dataset of SEM images on Ag-Bi-I thin films, excluding low-quality defocused images. Each of these 141 SEM images was then divided into 4 × 4 patches, resulting in a total of 2256 patches. Additionally, 146 full SEM images were also included in the training dataset. A key point detection algorithm, as detailed in Methods section, was employed in defect-prone regions to categorize images as “defective” or “non-defective”, with the identified key points represented by green dots in Fig. 1b. This approach provided an effective alternative to manual labeling. Three neural network architectures were evaluated for their classification performance. VGG16, a 16-layer deep neural network developed by the Visual Geometry Group, features a straightforward architecture, as shown in Fig. 1b^31^. VGG16 was able to extract hierarchical features and outperformed the other two models when fine-tuned for our dataset, achieving an accuracy of 97% on the test set, as shown in Fig. 1c. The results for the other two neural networks, ResNet50 and MobileNet, can be found in Fig. S2.

By integrating Daisy segmentation, classification, and clustering models in this study, the Daisy Image Interpreter was configured to output two numerical metrics from each SEM image: the overall defect percentage and a distribution of individual grain areas. These metrics were selected due to their critical importance for optimizing Ag-Bi-I semiconductor film morphology. Our demonstration aims to highlight examples of the information that can be extracted using the Daisy Image Interpreter. As shown in Fig. 2, we present two examples of defective SEM images processed by the Daisy Image Interpreter, showcasing the framework’s flexibility in handling SEM images across different magnifications. In practice, the framework can be readily adapted to extract additional grain statistics as needed, as demonstrated in Fig. S3, where we show the distribution of equivalent grain radii. Notably, Daisy processes each of these images in ~30 s, making it far more efficient than manual counting, which takes roughly an hour, and 120 times faster than state-of-the-art manual grain size characterization using X-ray diffraction (XRD), which requires about an hour for measurements and Pawley fitting to determine grain sizes. Furthermore, when we attempted to segment Fig. 2c using ImageJ^32^, the process was also significantly slower, taking around 25 min, and the results were notably inferior to those produced by the Daisy Image Interpreter, as shown in Fig. S4a, further underscoring the necessity of our model for accurate and reliable image segmentation.

### Processing – microstructure relationships

**Fig. 3:**
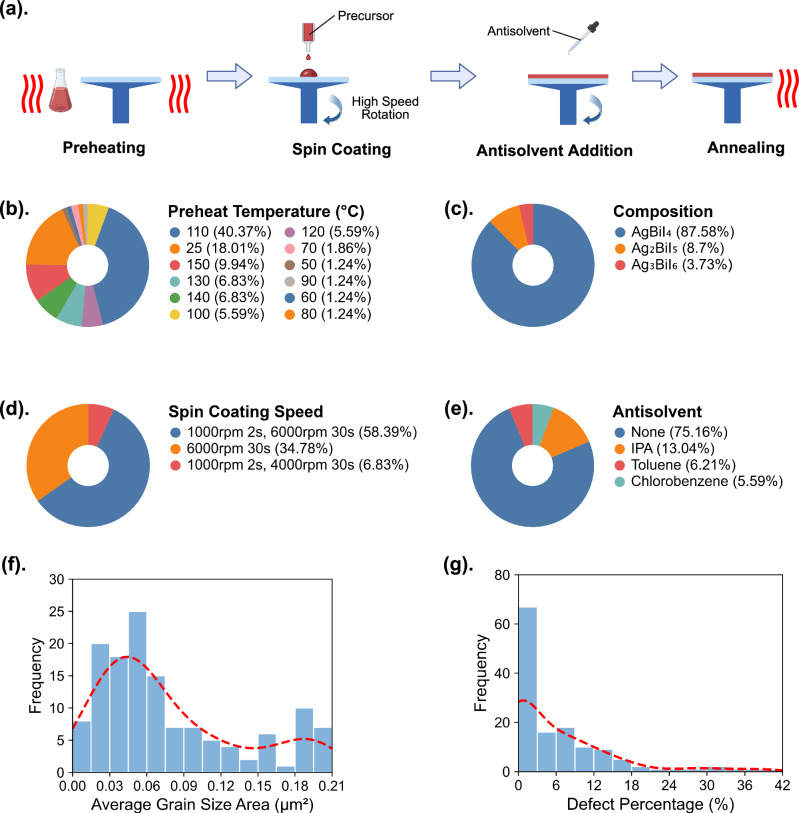
Overview of the full dataset analyzed in this study. **a** Schematic representation of the multi-step processing of thin film semiconductors via spin coating inside a N_2_-filled glovebox. **b**–**e** The preheat temperature (for both substrate and precursor), composition, spin coating speed, and antisolvent used in this study. A temperature of 25 °C indicates that no preheating was employed. **f**, **g** Distribution of average grain size area and defect percentage, extracted using Daisy image interpreter, with the red dashed line representing the kernel density estimation of the distribution.

The microscopy dataset used in this study to train the Daisy Image Interpreter consists of historical images collected over several months by three of the authors at the University of Oxford following the four processing steps of substrate and percussor preheating, spin coating, antisolvent addition, and annealing, as illustrated in Fig. 3a. Figure 3b–e show the diverse range of synthetic conditions of each processing step employed, leading to the formation of various microstructural features observed in the 161 SEM images in this study. Each image captures a spin-coated Ag-Bi-I thin film sample, synthesized by one of the 27 distinct combinations of synthetic conditions across three compositions, AgBiI_4_, Ag_2_BiI_5_ and Ag_3_BiI_6_. Detailed spin coating parameters used in the synthesis of Ag-Bi-I thin-film semiconductors can be found in the GitHub repository linked to this manuscript. Figure 3b shows that the most frequently used preheat temperatures for both substrate and precursor, each exceeding 10% of the total samples, were 110 °C and 25 °C. Figure 3c illustrates the compositions of the fabricated thin films, with the majority of efforts focusing on AgBiI_4_. Figure 3d details the spin coating speeds, with the combination of 1000 rpm for 2 s followed by 6000 rpm for 30 s being the most common protocol. Figure 3e outlines the antisolvents used, showing that while most samples were synthesized without an antisolvent, a substantial subset employed isopropyl alcohol (IPA), toluene, or chlorobenzene. This diversity in antisolvent usage allowed for an examination of its impact on grain boundary formation and defect modulation.

Using the Daisy Image Interpreter, we extracted the grain area distribution and defect percentage for each thin-film sample from the 161 SEM images. Although the grain area histograms in Fig. 2b, d exhibit a moderate right skew, the arithmetic mean remains a reliable descriptor since it accounts for every data point in the distribution and is widely used in the literature to correlate grain size with optoelectronic performance^33–35^. Accordingly, we calculated the average grain area for each sample and added this metric together with the defect percentage to our dataset. The distributions of these values are illustrated in Fig. 3f, g, and the relationship between these metrics and each synthesis parameter can be visualized in Fig. S5. Most samples showed an average grain size area ranging from 0.02 µm^2^ to 0.06 µm^2^ and exhibited minimal defect percentages. However, some display substantially larger grain sizes, reaching up to 0.22 µm^2^, or higher defect percentages, exceeding 30%, which underscores the variability in film quality throughout the dataset. This variation highlights the critical importance of optimizing synthesis parameters to consistently achieve high-quality thin-film solar cells.

### Daisy synthesis planner

**Fig. 4:**
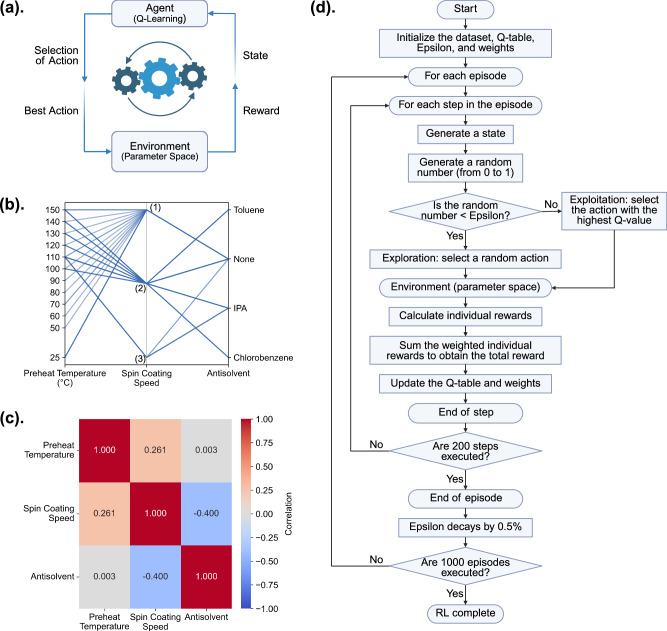
Reinforcement learning (RL) algorithm used for synthetic optimization. **a** Schematic illustration of the general concept of the RL algorithm. Specifically, this RL algorithm was used to identify the processing parameters of AgBiI_4_ that yield the largest grain size and no pinholes. In this study, only samples with a composition of AgBiI_4_ were considered within the parameter space, so the composition parameter was not included in the optimization process. **b** Material synthesis conditions used for optimization, with each connected line representing one combination of synthesis conditions in the dataset. In the “Spin Coating Speed” column, (1) represents 6000 rpm for 30 s, (2) represents 1000 rpm for 2 s then 6000 rpm for 30 s, and (3) represents 1000 rpm for 2 s then 4000 rpm for 30 s. **c** Correlation heatmap of the three synthesis steps employed for optimization. **d** Detailed workflow showing how the RL agent systematically explores the full combinatorial space of synthesis conditions to achieve optimization beyond the original dataset.

Identifying optimal conditions for each step of the solution process, from precursor and substrate preheating, spin coating speed setting, to antisolvent dripping, is fundamentally a multi-objective optimization problem. Two distinct challenges make such optimization challenging by direct learning from the historical data. First, unlike brute-force screening, manual experimental data collected tends to skew toward a limited set of synthesis conditions that are deemed promising based on human intuition. However, the overall search space is vast. For instance, with 12 different temperatures for both substrate and solution preheating respectively, 3 spin coating speeds, and 4 antisolvent-dropping conditions, there are 1728 possible parameter combinations to explore. Given that fabricating 10 films takes around an hour and acquiring 5 SEM images requires another hour, we can process synthesize and characterize 20 films per day on average. Additionally, scheduling the necessary equipment requires about a week in advance. At this rate, completing all experiments would take at least 87 weeks. Second, the heterogeneity in the data structure of synthetic conditions adds complexity to data analysis and decision-making. For instance, some experiments in the dataset involve a single spin coating speed, such as 6000 rpm for 30 seconds, while others employ a two-stage coating process, such as 1000 rpm for 2 s followed by 6000 rpm for 30 s (Fig. 3d). There is no simple way to numerically represent how one synthesis condition is different from another, which complicates pattern recognition and optimization problem framing. In this work, we formulated the processing parameter optimization in an offline RL setting, with the agent learning exclusively from a static, pre-collected dataset of synthetic conditions and microstructure metrics, treating each condition as categorical data. RL has increasingly been explored in chemistry and materials science for tasks ranging from composite design to predictive synthesis using simulated data. Sui et al. applied deep RL to automate materials design, where a deep Q-network (DQN) architecture was used to optimize composite structures based on finite element analysis simulations, achieving significant improvements over genetic algorithms^36^. In a recent study of growing monolayer MoS_2_ via chemical vapor deposition, an offline RL agent was trained on synthesis simulations to predict reaction conditions for phase-pure materials^37^. Despite these advances, RL has not yet been applied to optimize the microstructure of thin-film semiconductors trained on experimental data. As shown in Fig. 4a, b, in the Daisy Synthesis Planner, the RL agent iteratively interacts with the parameter space environment by selecting actions (i.e., synthesis conditions) from three processing steps: preheating, spin coating, and antisolvent addition, and receiving feedback via a reward function designed to evaluate film quality (see Methods section for details). We define the experimental parameter space as the environment for the agent to learn from, enabling it to search for optimal solutions as the agent identifies sets of conditions associated with high rewards. The primary parameter space, illustrated in Fig. 4b, includes the choices of the preheat temperatures of the substrate and precursors, spin coating speed, and antisolvent usage, which forms the design space for microstructure optimization. A model-free RL, deep Q-learning, was employed, which enables the agent to train within the experimental parameter space without requiring prior knowledge of the processing - microstructure relationships. Given that each target composition has a unique processing window, we focus on optimizing the film morphology of AgBiI_4_ as a case study for the development of the Daisy Synthesis Planner. The RL agent’s primary objective is to propose optimal processing windows that yield desirable microstructures, as defined by maximizing average grain sizes and minimizing the percentage of defect coverage. We used static weighting of grains (70%) and defects (30%), as assigned by chemists in our team based on domain experts. In addition, to assess the robustness of this weighting, we set two parallel RL agents using alternative weightings of 90%/10% and 50%/50% (grains/defects), with more details provided in Supplementary Information (Fig. S6 and Table S1).

Two distinct approaches are implemented for the RL agent: the first, serving as the baseline for implementing the exploratory RL algorithm, focuses on learning from existing synthesis condition combinations, treating the completion of three processing steps of preheating, spin coating, and antisolvent addition as a single action, which is illustrated in Fig. S7, while the second approach, the true exploration-focused implementation, explores new regions in parameter space, where each processing step is considered independently, as shown in Fig. 4d. In the RL baseline approach, the agent is trained using the existing experimental dataset of AgBiI_4_, evaluating only the parameter combinations explicitly shown in Fig. 4b. Rewards are assigned based on the quality of each set of synthesis conditions, allowing the agent to learn the processing-microstructure relationships by interacting with the existing combinations of synthesis conditions in the historical dataset. The exploratory RL algorithm extends this search by evaluating the contributions of each process step by step, in pursuit of new combinations of synthesis conditions. Unlike the RL baseline, which assigns an immediate reward based on a chosen set of preheat temperatures, spin coating speed and antisolvent selection, the exploratory RL employs a weighted sum of individual rewards for each processing step, enabling greater flexibility in generating new combinations of synthetic parameters. Figure 4c shows the correlations among synthesis parameters across different processing steps, demonstrating minimal interdependence, which supports the independent optimization of each parameter in the exploratory RL. In both approaches, the agent balances exploration and exploitation, updating a Q-table, a matrix mapping state-action pairs to expected rewards, at each episode to iteratively refine its decision-making strategy based on past experiences (see Methods for details on its calculation). In the exploratory RL, the agent further enhances this process by dynamically adjusting reward weights to optimize decision-making.

**Fig. 5:**
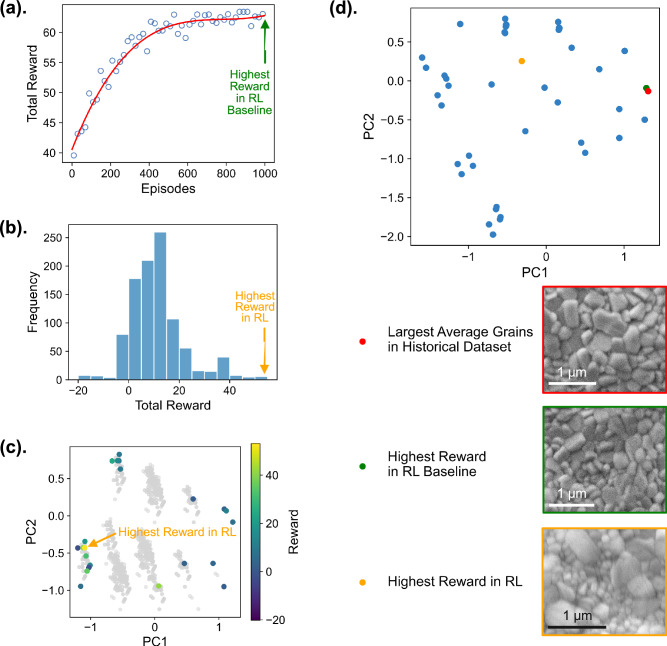
Optimization and validation of AgBiI_4_ synthesis conditions using the reinforcement learning (RL) algorithm. **a** Total reward progression as a function of episodes for the RL baseline (detailed in Fig. S7), demonstrating RL performance over time. The synthesis conditions are optimized as the reward peaks, indicating convergence. **b** Reward distribution across 1000 training episodes for the exploratory RL (described in Fig. 4d), where the optimal synthesis conditions align with the highest reward values. **c** Principal component analysis (PCA) visualization of all unique synthesis conditions explored by the RL agent over 1000 training episodes. Each point represents a unique condition, colored by its corresponding reward. Gray points in the background represent the full design space of 1728 possible parameter combinations, illustrating that the agent selectively explores a subset of this space during training. **d** Comparison and validation of the best synthesis conditions identified by both the RL baseline and exploratory RL against the historical dataset’s top-performing condition with the largest average grains detected. The upper panel visualizes synthesis conditions in the parameter space, analyzed via PCA on the original full dataset containing all three compositions: AgBiI_4_, Ag_2_BiI_5_, and Ag_3_BiI_6_. The lower panel presents the scanning electron microscopy (SEM) images of solar cells fabricated with the respective synthesis parameters, and their average grain sizes are specified in Table 1. To facilitate direct visual comparison, the SEM image for the “Highest Reward in RL”, originally acquired at a different magnification, was cropped and rescaled to match the scale of the other two images.

The results of the two approaches implemented by the RL agent are summarized in Fig. 5. Figure 5a highlights the progression of total rewards across episodes for the RL baseline, where the stabilization of rewards over iterative optimization indicates convergence after 1000 episodes. The optimization outcome closely aligns with the historical dataset’s best sample identified by the Daisy image interpreter, exhibiting only minor deviations (see Table 1), which confirms the agent’s ability in identifying optimal sets of synthesis combinations. In contrast, the exploratory RL explores a broader design space, identifying new optimal synthesis conditions at each processing step beyond the existing combinations. Since the progression of it exhibits an oscillatory pattern, likely due to the limited dataset of only 27 distinct combinations compared to the 1728 possible ones, the reward distribution is plotted in Fig. 5b, and the RL agent’s 1000-episode exploration of the design space is visualized via principal component analysis (PCA) for dimension reduction in Fig. 5c. These visualizations highlight the discovery of parameter combinations yielding the highest rewards, a process that takes about 3.5 min by the RL agent operated on Google Colab. To visualize the optimal processing windows identified by both approaches, we also performed a second PCA on the full parameter dataset encompassing all three compositions, AgBiI_4_, Ag_2_BiI_5_, and Ag_3_BiI_6_, as shown in Fig. 5d and Fig. S8. The optimal processing window identified by the RL baseline, which consists of three synthesis conditions from the preheating, spin coating, and antisolvent addition steps respectively, are close to the top-performing window in the historical dataset in the reduced two-dimensional space, which yielded the largest average grains detected experimentally. The exploratory RL, on the other hand, uncovers a significantly different set of synthesis conditions leading to the suggestion of a new processing window in Table 1. Our results demonstrate RL’s ability to explore new regions of parameter space and identify novel synthesis pathways.

**Table 1:** Validation of the reinforcement learning (RL) algorithm, which presents the optimization results of the RL baseline and exploratory RL, comparing them to the best sample with largest average grains observed within the historical dataset

	Preheat Temperature (°C)	Composition	Spin Coating Speed	Antisolvent	Average Grain Size Area (μm^2^)	Visible Pinhole (%)
Largest Average Grains in Historical Dataset	110	AgBiI_4_	1000 rpm 2 s, 6000 rpm 30 s	Toluene	0.0713	0
Highest Reward in RL Baseline	110	AgBiI_4_	1000 rpm 2 s, 6000 rpm 30 s	Chlorobenzene	0.0700	0
Highest Reward in RL	140	AgBiI_4_	6000 rpm 30 s	None	0.0697	0

Listed are the synthesis conditions with corresponding average grain sizes and defect percentages by Daisy image interpreter. The preheat temperature is the same for both substrate and precursor.

To validate the new processing window suggested by RL, a new round of synthesis experiments was conducted using the AI-suggested optimal conditions. Two independent AgBiI_4_ films were fabricated at the optimal conditions, and three SEM images were acquired for each sample. A representative SEM image, shown at the bottom of Fig. 5d, was compared to the films produced under the best synthesis conditions identified in the training dataset. Qualitatively, all three SEM images show similar grain sizes and minimal visible defects. Quantitative analysis using the Daisy image interpreter confirmed that all three synthesis conditions produced films with nearly identical grain area distributions (Fig. S9). Moreover, the newly fabricated AgBiI_4_ film exhibited no detectable pinholes and an average grain size comparable to the top-performing samples in the historical dataset. These results are summarized in Table 1 and Table S2, which highlights that the new set of synthesis conditions proposed by RL achieves pinhole-free, large grain films, thereby demonstrating the utility of this agentic approach in synthesis planning based on historical data. Considering that fabricating and imaging the optimal sample takes an additional 2 h, and scheduling the equipment requires about a week, this validation process of RL was 87 times faster than the brute-force synthetic screening over the 1728 possible synthesis conditions. If we exclude the equipment scheduling and waiting time and assume continuous experimental operations, the difference in the experimental time needed between RL one-shot learning and the manual synthetic screening exceeds 500-fold, highlighting the significant potential of the Daisy framework in accelerating microstructure development for thin films.

## Discussion

In this study, we integrated computer vision techniques with an offline RL agent to intelligently optimize synthesis parameters based on microstructural feedback from a historical dataset. Unlike traditional approaches that treat these tasks separately, the integration of automated image analysis and synthesis planning into a single framework exemplifies assembling compound models into an AI agent with greater autonomy and decision-making capability, allowing the adaptability and efficiency in the microstructure optimization process. The multi-step process optimization in this context is challenging due to the nature of the training dataset, which includes categorical parameters and inherent biases. Specifically, synthesis parameters, such as preheat temperature, spin coating speed, and antisolvent type, as illustrated in Fig. 4b, are categorical, lacking intrinsic numerical relationships. This complexity renders traditional gradient-based methods unsuitable. The dataset is significantly imbalanced, with certain parameter combinations overrepresented. For example, spin coating speeds like “6000 rpm for 30 s” occur far more frequently than other combinations, while the antisolvent column is heavily skewed toward “None”, with limited diversity in other solvents. This imbalance restricts the generalizability of predictive models, as they are trained on a narrow and biased distribution of parameter combinations, limiting their ability to extrapolate to underrepresented conditions. In this section, we discuss several methods that could potentially be used for the optimization task, and by comparing them, we found RL achieves superior results, as presented in the previous section.

One common approach for machine-learning-guided design of experiment has been Bayesian optimizaiton (BO), which leverages surrogate models to balance exploration and exploitation^38^. BO can handle categorical variables by encoding them as discrete inputs and iteratively refines its exploration of the parameter space^39^, making it more efficient than exhaustive search methods. However, the effectiveness of BO is heavily dependent on the quality of the surrogate model^40^. Gaussian processes, commonly used in BO, are well-suited for handling uncertainty but struggle with interdependencies between parameters and imbalanced datasets^41,42^, as the case in this study, shown in Fig. S10. Given the limitations of Gaussian processes in such contexts, identifying a suitable surrogate model is a nontrivial and resource-intensive task, further constraining BO’s applicability here. Meanwhile, while tree-based methods such as Random Forests (RF) offer an alternative by naturally accommodating categorical variables^43,44^, their performance is highly sensitive to data imbalances^45^, with the results shown in Fig. S11 highlighting significant discrepancies in our attempts to model the average grain size area and defect percentage using RF, as further discussed in the Supplementary Information. In this study, RL’s model-free architecture eliminates the need for surrogate model selection, as required in BO^46^. Through iterative learning, RL adapts to the environment represented by the dataset and explores underrepresented parameter combinations using an ε-greedy strategy^47,48^. This exploration mitigates the effects of dataset imbalance, allowing the RL agent to consider rare but impactful parameter combinations during training. By incorporating a reward mechanism based on defect percentage and grain size at the same time, the framework ensured these rare combinations were not overlooked. Moreover, as shown in Fig. 5a, RL’s loop structure enables cumulative optimization, allowing decisions to adapt dynamically based on earlier results and prioritizing parameters with the greatest impact on the quality of material synthesis. Although RL is typically applied to large datasets and can become unstable or overfit when training samples are sparse^49,50^, our study still demonstrates its effectiveness with fewer than 200 samples, a feasibility also validated by Qin et al.^51^. Notably, Q-learning’s flexibility and model-free approach make it suited for optimization tasks in this data-limited environment^52^. This is supported by prior work from Kaliappan et al. where Q-learning effectively managed home energy consumption by dynamically learning optimal appliance usage policies without requiring a predefined model, demonstrating its adaptability to complex, real-time decision-making scenarios^53^. Nevertheless, because our RL agent was trained on a relatively sparse dataset, it has not fully converged to the deterministic optimum and its generalizability to untested regions remains uncertain; instead, its chief merit in this context is the ability to propose novel synthesis conditions outside the previously explored space and was able to achieve similarly high rewards. Future investigations are expected to focus on expanding the experimental dataset broadening the agent’s applicability.

In summary, we developed Daisy, a computational framework that serves as a key component of future fully autonomous self-driving laboratories, consisting of compound AI models to collectively learn from historical visual microscopic images and propose new synthesis conditions for Ai-Bi-I perovskite-inspired thin-film semiconductors. Daisy’s image interpreter achieved 97% in defect defection accuracy and over 120× acceleration in extracting grain and pinhole information from SEM images compared to manual methods, followed by an additional 87× acceleration in synthesis planning compared to brute force screening, by using an offline RL agent to suggest next experiments from historical synthesis and characterization data. Overall, as self-driving labs evolve with ever more sophisticated robotic workflows and real-time analytics, Daisy stands poised to accelerate the development of high-efficiency indoor photovoltaics across a wider range of compositions, and the AI image interpreter and synthesis planner developed in this study can be readily applied to other material classes.

## Methods

### Thin-film synthesis

AgBiI_4_ thin films were prepared in a nitrogen-filled glovebox by dissolving silver iodide (AgI) powder (99.98%, Sigma-Aldrich) and bismuth iodide (BiI_3_) (99.9%, Alfa Aesar) in a 1:1 molar ratio using dimethyl sulfoxide (DMSO, anhydrous, 99.99%, Sigma-Aldrich) as the solvent. The solution was stirred overnight at 70 °C and then filtered through a 0.2 µm polytetrafluoroethylene (PTFE) filter (Fisher Scientific).

For Ag_2_BiI_5_ and Ag_3_BiI_6_ thin films, AgI powder (Premion®, 99.999%, Thermo Scientific) and BiI_3_ (Puratronic®, 99.999%, Thermo Scientific) were mixed without further purification. To prepare a 34% (weight ratio) solution, DMSO (anhydrous, ≥99.9%, Sigma-Aldrich) and N, N-dimethylformamide (DMF, anhydrous, 99.8%, Sigma-Aldrich) were added in a 10:1 volume ratio. The solution was stirred for 2 h at 70 °C and filtered with the same PTFE filter as above.

Before spin coating, glass substrates were cleaned through sequential sonication in deionized (DI) water, acetone, and isopropanol, followed by UV-ozone treatment (ATG Scientific, UVC1014) for 15 min after each step to ensure thorough surface preparation. After that, both the solution and substrates were preheated to the temperatures specified in the dataset using a stirring hotplate (Radleys TECH). A 200 μL aliquot of the solution was then pipetted onto the substrate and spin coated. For certain thin films, 1 mL of an antisolvent, either chlorobenzene (anhydrous, 99.8%, Merck), IPA (99.5%, Sigma-Aldrich), or toluene (anhydrous, 99.8%, Sigma-Aldrich), was dripped onto the substrate 20 seconds into the second stage of spin coating using a 1 mL pipette. The final thin films were annealed at the designated temperature and time as per the dataset.

### Scanning electron microscopy (SEM)

SEM imaging was conducted using a Zeiss Merlin - Analytical system, performed at an electron energy of 10 keV, with the chamber pressure maintained at approximately 10^−5 ^Pa. Depending on the experiment, some samples were grounded using carbon tape or silver paste to minimize charge accumulation. Slight charge buildup and sample drift were observed in ungrounded samples. No conductive coating was sputtered onto the thin films before SEM imaging.

### Data preprocessing of SEM images

To ensure consistency and relevance in the analysis, a comprehensive preprocessing pipeline was implemented for the SEM images. This pipeline encompassed file renaming, metadata extraction, and image cropping to standardize the data and isolate regions of interest. The SEM image dataset, acquired under varying experimental conditions, was organized into distinct folders reflecting synthesis parameters. A Python-based automation script was developed to trace these parameters systematically by parsing folder names. Metadata, including critical synthesis parameters such as spin coating speed, temperature, chemical composition, and antisolvent, were extracted using a rule-based mapping algorithm derived from predefined associations provided with the dataset.

To focus the analysis on pertinent regions, the bottom 10% of each image, typically containing extraneous background artifacts, was cropped. This operation was carried out using the Python Imaging Library (PIL)^54^, ensuring that the original aspect ratio was preserved while standardizing the field of view across all samples. These preprocessing steps facilitated a uniform and targeted dataset for subsequent analysis described in the manuscript.

### Segment Anything model (SAM)

SAM, developed by Meta, was utilized to achieve precise and automated segmentation of SEM images, eliminating the need for manual intervention^28^. A pre-trained Vision Transformer (ViT) model (using the vit_l configuration) was employed, executed on a Google Colab A100 GPU to leverage advanced hardware acceleration for computational efficiency.

The initial implementation used SAM’s default configuration with standard model parameters designed for general-purpose segmentation tasks, establishing a baseline for segmentation quality and serving as a benchmark for further improvements. SEM images were first converted to RGB format using the OpenCV package to meet SAM’s input requirements^55^. The model then generated segmentation masks for each image, which were visualized by overlaying semi-transparent masks on the original images. To improve interpretability, the visualization function sorted masks by area to prioritize larger regions, assigned random colors for differentiation, and presented them as overlays, as shown in the red block in Fig. 1a.

To enhance precision, specific model parameters, as outlined in Table 2, were later optimized using a random search approach, mirroring the structured methodology of the initial implementation. This iterative refinement ensured robust segmentation performance across diverse image features, providing a solid foundation for downstream analyses and insights.

**Table 2:** Parameters used for fine tuning of the Segment Anything Model (SAM), along with their corresponding descriptions and implications

Parameter	Description	Implication
points_per_side	Number of sampling points along each side of the image grid.	Higher values improve granularity but increase computation.
pred_iou_thresh	Minimum IoU threshold for mask predictions.	Stricter thresholds (e.g., 0.7–0.9) enhance precision but may exclude valid masks.
stability_score_thresh	Filters masks based on their stability across scales.	Higher values (e.g., 0.9–0.95) favor stable masks but risk discarding borderline regions.
crop_n_layers	Number of hierarchical cropping layers for segmentation.	More layers improve segmentation of small objects but increase processing time.
crop_n_points_downscale_factor	Downscales the number of points sampled in cropped regions.	Smaller factors preserve resolution but increase computational cost.
min_mask_region_area	Minimum size of mask regions	Filters small noisy masks. Set this based on object size in your dataset (e.g., around 0.1–1% of image resolution).

### Key point detectors

To automate the identification of regions exhibiting specific morphological features, such as pinholes and gaps, a key point detection methodology was developed to replace manual labeling efforts in SEM image analysis. The images were first processed in grayscale to emphasize intensity-based features, which are essential for detecting morphological variations. A global thresholding technique was then applied to isolate darker regions, which are often indicative of potential defects. The threshold values were dynamically determined based on the overall intensity and brightness characteristics of each SEM image. Following thresholding, the Harris Corner Detector was employed to identify key points within the images^56^. The resulting response map was normalized, and key points were extracted by applying a threshold to the normalized response. These key points, which represent regions of interest, were visualized as green dots overlaid on the original grayscale images, as depicted in Fig. 1b. In the final step, images with extracted key points were labeled as defective, while those without were categorized as non-defective.

### VGG16 classification algorithm

The VGG16 architecture, pre-trained on ImageNet, was utilized as the backbone for the classification task^31,57,58^. As depicted in Fig. 1b, custom layers were integrated on top, consisting of a global average pooling layer, a dense layer with 256 neurons and rectified linear unit (ReLU) activation, and a dropout layer with a rate of 0.5. The output layer featured a single neuron with a sigmoid activation function, designed for binary classification (between “with defects” and “no defect”). During the initial training phase, the VGG16 base layers were frozen to leverage pre-trained weights, minimizing training time and reducing the risk of overfitting. Only the custom layers were trained at this stage, achieving a validation accuracy of 95.83% in the early epochs. To further enhance performance, the base layers of VGG16 were unfrozen, allowing fine-tuning across the entire network. The model was recompiled with a reduced learning rate of 1 × 10^−5^ to retain pre-trained features while adapting to our SEM dataset. This fine-tuning step improved the model’s generalization, yielding a test accuracy of 97.1% with a test loss of 0.121, demonstrating a robust and well-calibrated classifier.

### Daisy clustering algorithm

K-means clustering was applied to the segmented images to classify regions into defects and grains. For each image, the mean intensity of pixels within each segmentation mask was computed by applying the mask to the original grayscale SEM image. These mean intensities formed the feature set for clustering. Using the K-means algorithm from the scikit-learn library^59^, the segmentation masks were grouped into two clusters, corresponding to darker regions (defects) and lighter regions (grains), aligning with the goal of distinguishing between defective and grain areas.

Furthermore, to eliminate redundant representations, overlapping masks were evaluated using the IoU metric. Masks with an IoU exceeding 0.5 were considered redundant, and in such cases, the larger mask (based on area) was retained, while the smaller overlapping masks were discarded. This step ensured that each region was represented by a unique, non-redundant mask.

The lighter masks, classified as grains, underwent additional filtering. Masks occupying more than 10% of the total image area were excluded, based on domain-specific knowledge of typical grain sizes. Finally, the refined masks were visualized (as shown in the yellow block in Fig. 1a) to verify their spatial alignment with observed defects and ensure accurate representation of the regions of interest.

### Reinforcement learning (RL) algorithm

A Q-learning-based offline RL algorithm was employed to optimize the synthesis conditions for spin coating AgBiI_4_ thin films, using two distinct approaches illustrated in Fig. 4d and Fig. S7. The RL agent was trained in 1000 episodes, with each episode consisting of 200 steps corresponding to parameter adjustments. At the beginning of each step, the training initiated from a random state within the unexplored parameter space of the current episode. The agent followed an ε-greedy exploration strategy, where, with probability ε, it explored new parameter sets by selecting a random action, and with probability 1−ε, it exploited its learned policy by selecting the action expected to maximize the reward. For the RL baseline, the action modified the combination of multiple parameters simultaneously, while for the exploratory RL, the action adjusted one parameter at a time. After performing the action, the agent transitioned to a new state and evaluated the reward associated with this transition.

The reward calculation ensured a balanced trade-off between the objectives of maximizing grain size and minimizing defect coverage by applying normalization to scale the raw values of these metrics into the range [0, 1] following Eq. (1).

1 $${x}_{{normalized}}=\frac{x-{x}_{\min }}{{x}_{\max }-{x}_{\min }}$$

And the reward was then calculated as

2 $$r=0.7\times {\left({grain\; size}\right)}_{{normalized}}-0.3\times {\left({defect\; percentage}\right)}_{{normalized}}$$

For the RL baseline, the reward calculated by Eq. (2) was directly used as the final reward for the step. For the exploratory RL, Eq. (2) was applied to evaluate the reward for each individual parameter, and a weighted summation of these rewards was computed as

3 $${r}_{{total}}=\sum _{i}{w}_{i}{r}_{i}$$

Where ${w}_{i}$ represents the weight associated with each parameter, which was dynamically updated after every step.

Afterwards, the Q-value for each state-action pair was updated in the Q-table using the Bellman equation

4 $$Q\left(s,a\right)=Q\left(s,a\right)+\alpha \left(r+\gamma \max Q\left({s}^{{\prime} },{a}^{{\prime} }\right)-Q\left(s,a\right)\right)$$

Where $s$ and $a$ represent the current state and action, $Q\left({s}^{{\prime} },{a}^{{\prime} }\right)$ represents the estimated future reward for the next state-action pair, $\alpha$ is the learning rate, and $\gamma$ is the discount factor. Simultaneously, the weight ${w}_{i}$, which was crucial for the reward calculation in the exploratory RL, was dynamically adjusted to prioritize parameters with greater influence on the reward. This adjustment was based on

5 $${w}_{i,{new}}={w}_{i,{old}}+\eta \left(\frac{{E}_{i}}{{\sum }_{i}{E}_{i}}-{w}_{i,{old}}\right)$$

where $\eta$ was the learning rate for weight adjustment, and ${E}_{i}$ quantifies the impact of the $i$^th^ parameter on the reward, defined as

6 $${E}_{i}=\left|{r}_{i}-{r}_{{total}}\right|$$

After every episode, the exploration rate ε in the ε-greedy strategy decayed by 0.5%, encouraging the agent to shift from exploration to exploitation as training progressed.

### Principal Component Analysis (PCA)

The PCA analysis is implemented using the PCA class from scikit-learn^59^, with categorical variables first encoded via one-hot encoding using scikit-learn’s OneHotEncoder. This transforms categorical variables into a binary matrix, where each category is represented as a unique binary vector. The high-dimensional binary data is then processed through PCA, which involves several steps: mean centering by subtracting the mean of each feature to prevent bias, computing a covariance matrix to measure feature relationships, performing eigen decomposition to obtain eigenvalues (representing variance) and eigenvectors (defining principal axes), and projecting the data onto the top k eigenvectors. In our case, the data is reduced to two principal components, PC1 and PC2, as shown in Fig. 5c, d, which preserves key patterns while filtering out noise, providing an interpretable 2D representation of the high-dimensional data.

## Supplementary information

Supplementary Information

## Data Availability

All image data used in this manuscript are available from the Oxford Research Archive (10.5287/ora-ovqa54yn1).

## Code availability

The code used in this study together with all image support data (in Excel files) are openly accessible on GitHub (https://github.com/uwsunlab/Daisy.git).
