# Supplementary material for: Data-driven microstructural optimization of Ag-Bi-I perovskite-inspired materials
Source: NPJ Comput Mater. 2025 Jul 3;11(1):210. doi: 10.1038/s41524-025-01701-7 (PMC12226340; doi:10.1038/s41524-025-01701-7)
Supplement: Supplementary file 1 — Supplementary Information [file 41524_2025_1701_MOESM1_ESM.pdf]

Data-driven microstructural optimization of Ag-Bi-I perovskite-inspired materials

Kshithij Mysore Nandishwara,[1] Shuan Cheng,[1] Pengjun Liu,[2] Huimin Zhu,[2,3] Xiaoyu Guo,[2] Fabien C.-P. Massabuau,[3] Robert L.Z. Hoye,[2,*] Shijing Sun[1,*]

[1]Department of Mechanical Engineering, University of Washington, Seattle, WA 98105, USA
[2]Inorganic Chemistry Laboratory, Department of Chemistry, University of Oxford, South Parks Road, Oxford OX1 3QR, UK
[3]Department of Physics, SUPA, University of Strathclyde, Glasgow G4 0NG, UK
*Correspondence: shijing@uw.edu, robert.hoye@chem.ox.ac.uk

Supplementary Information

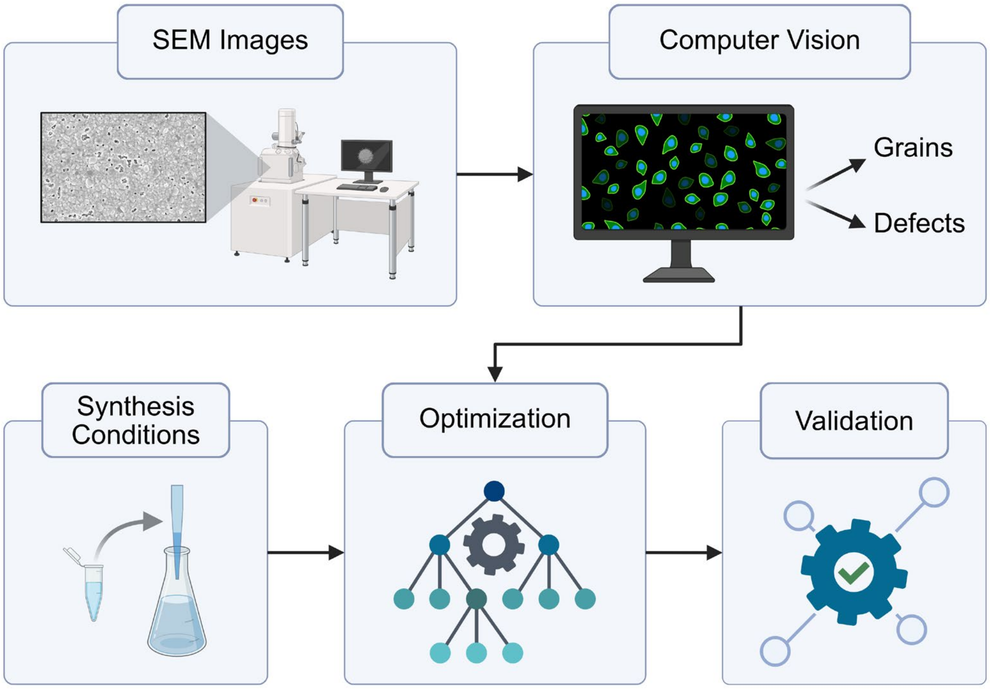

Figure S1: Schematic illustration showing the workflow of the study conducted in our paper. It starts with the original scanning electron microscopy (SEM) images to the validation of the best sets of synthesis conditions.

ResNet50 for classification

In addition to VGG16 shown in the main text, we explored ResNet50 (a Residual Network with 50 layers) for our classification task.[1] This state-of-the-art deep learning model addresses the vanishing gradient problem inherent in deep networks through residual connections. These connections enable ResNet50 to learn complex hierarchical representations effectively, making it highly robust for intricate classification tasks. Its depth and ability to capture subtle feature hierarchies are particularly advantageous for datasets with rich and complex patterns. However, these benefits come at the cost of increased computational demands. On our relatively small dataset, ResNet50 achieved a test accuracy of 73%, as illustrated in Figure S2(a).

MobileNet for classification

We also evaluated MobileNet, a model designed for efficiency in resource-constrained environments.[2] By utilizing depth wise separable convolutions, MobileNet significantly reduces computational requirements and model size, making it well-suited for applications prioritizing lightweight architectures and faster inference times. While not as inherently powerful as deeper models, MobileNet excels in scenarios with limited computational resources. On our dataset, it delivered a test accuracy of 96%, as shown in Figure S2(b).

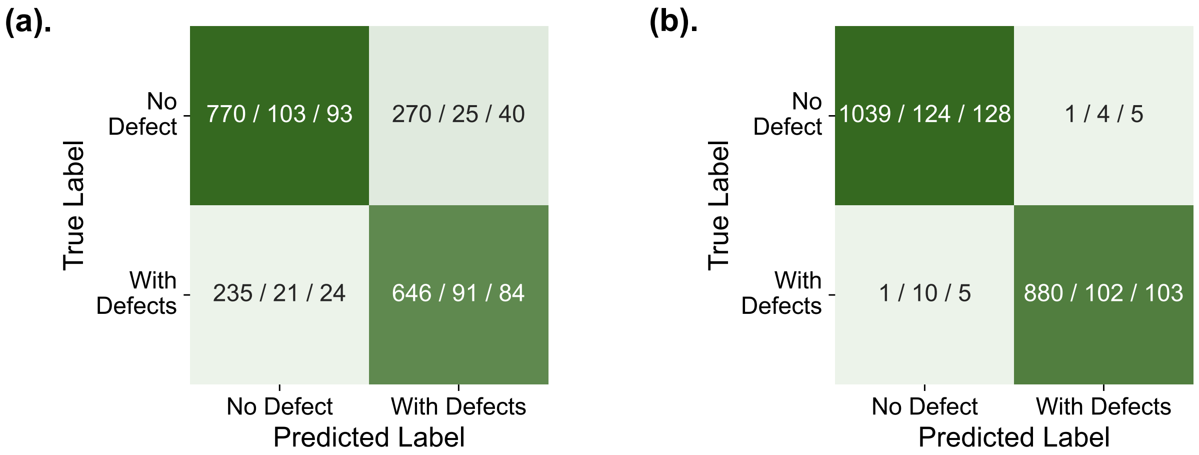

Figure S2: The confusion matrices for (a), ResNet50 and (b), MobileNet classification algorithms. For evaluation, some low-quality images were removed, and each image was divided into multiple patches to expand the dataset. Each entry in the confusion matrix is represented in the format of A / B/ C, where A is the performance on the training set (80% of data), B is on the validation set (10% of data), and C is on the test set (10% of data).

Equivalent grain radius distributions for representative samples

Figure S3 provides supplementary results on the equivalent grain radius distributions for the representative samples shown in Figures 2(a) and 2(c). These distributions exhibit similar shapes to the grain area distributions presented in Figures 2(b) and 2(d), and are derived using the following equation to calculate the equivalent radius:

$$R_{eq} = \sqrt{\frac{A}{\pi}} \tag{1}$$

Where A is the grain area.

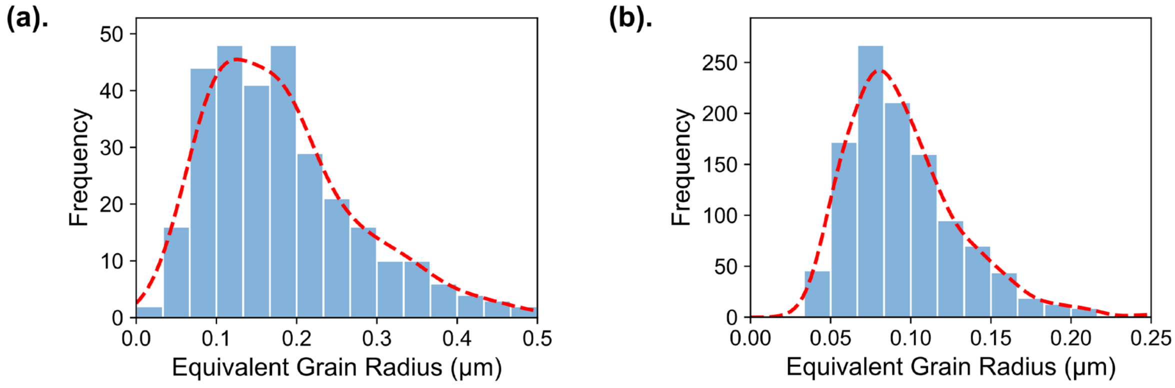

Figure S3: Equivalent grain radius distributions of representative results from the Daisy Image Interpreter. (a), The distribution corresponding to the thin film in Figure 2(a), with the red dashed line indicating the kernel density estimation of the distribution. (b), The distribution corresponding to the thin film in Figure 2(c), with the red dashed line indicating the kernel density estimation of the distribution.

Comparison to ImageJ on image segmentation

To benchmark ImageJ's segmentation against our Daisy Image Interpreter, we first created a ground-truth baseline by manually annotating grains and defects and then applying the LABKIT plugin's segmentation tool in ImageJ.[3,4] The initial output exhibited severe over-segmentation, blurred grain boundaries, and frequent misclassification of defects as grains. With multiple rounds of human-assisted refinement by making more manual annotations, ImageJ's results improved markedly, as shown in Figure S4(a). However, when compared to the fully automated output of the Daisy Image Interpreter in Figure S4(b), many adjacent grains remained unseparated. This persistent gap underscores the value of a domain-specific interpreter for precise and reliable grain-and-defect segmentation. Moreover, the manual annotation and refinement process for this image required approximately 25 min, significantly longer than the Daisy Image Interpreter's 38 s runtime.

(a). (b).

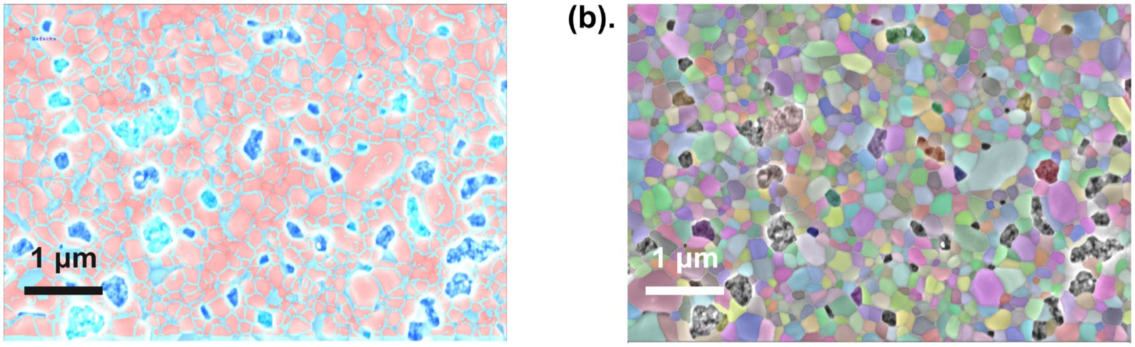

Figure S4: Segmentation performance comparison between ImageJ (using LABKIT plugin) and the Daisy Image Interpreter. (a), Manual annotation followed by iterative LABKIT segmentation in ImageJ (around 25 minutes in total), showing residual grain clumping and misclassified defects despite refinement. (b), Fully automated segmentation by the Daisy Image Interpreter (38 seconds runtime), with clear grain boundaries and accurate defect separation.

Synthesis conditions and grain characteristics

Complementing Figure 3(b) ~ (g), which individually illustrates various synthesis conditions and the distribution of grain characteristics, Figure S5 provides an integrated analysis linking synthesis conditions to grain characteristics. This figure visualizes the relationship between specific synthesis parameters and the resulting grain properties within our dataset, providing a clearer understanding of how parameter variations influence grain size and defect percentage. For instance, subplot (b) reveals that thin films fabricated at higher spin coating speeds tend to exhibit larger grain sizes, illustrating the role of this parameter in shaping microstructural outcomes.

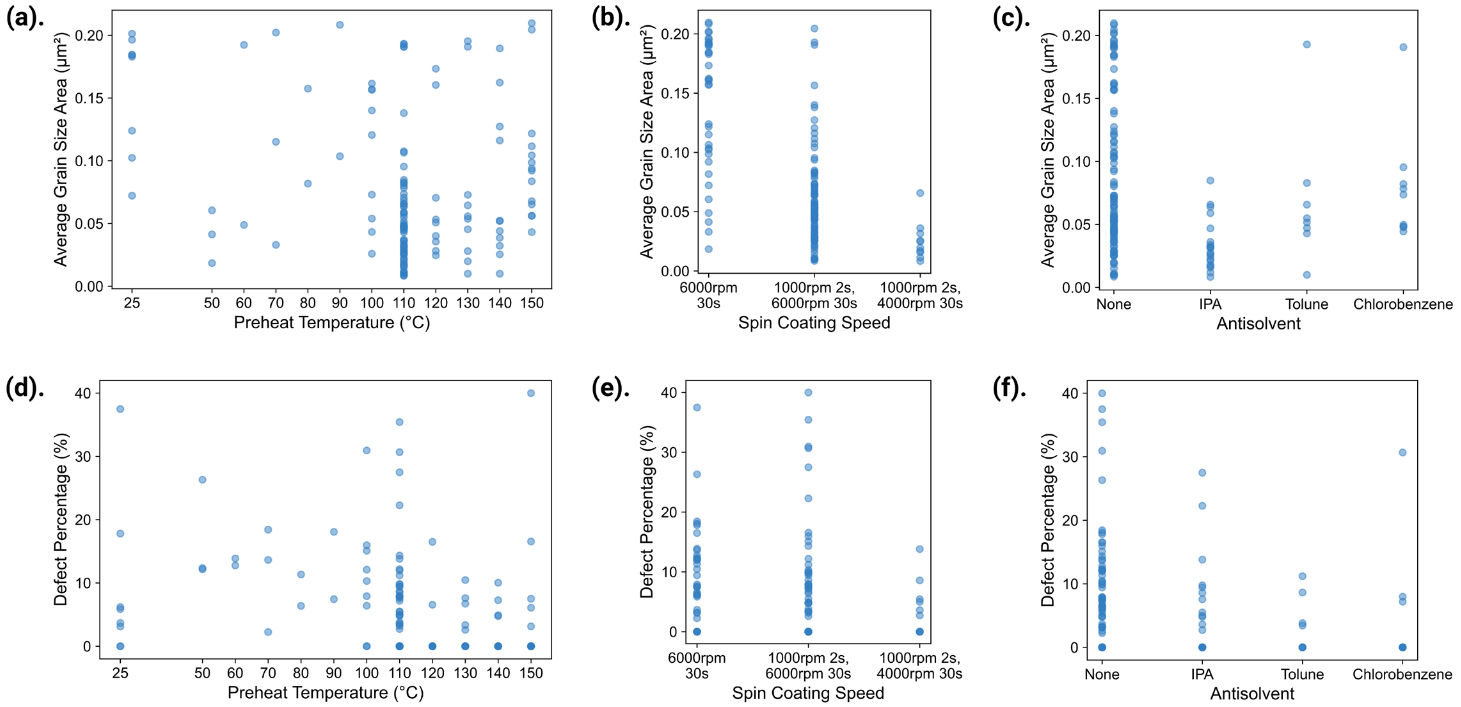

Figure S5: Scatter plots illustrating the variation in synthesis conditions and grain characteristics within the optimization dataset. Panels (a)-(c) depict the distribution of average grain size area for varying preheat temperatures, spin coating speeds, and antisolvents. Panels (d)-(f) show the distribution of defect percentages for these same synthesis parameters.

Additional information on reinforcement learning (RL) optimization

In the main manuscript, a 70% grains / 30% defects reward split was adopted based on expert guidance from our chemistry team. To assess the sensitivity of this choice, two additional RL agents were trained using alternative reward allocations of 90% / 10% and 50% / 50% (grains / defects). As shown in Figure S6 and Table S1, the 50% / 50% configuration produced optimal synthesis conditions nearly identical to those of the 70% / 30% baseline, demonstrating that a moderate increase in defect emphasis does not materially alter the agent's recommendations. In contrast, the 90% / 10% weighting yielded a markedly different set of top-performing conditions, indicating that extreme underweighting of defect metrics can substantially shift the optimization outcome. These results confirm that the 70% / 30% weighting scheme is robust, unbiased, and well-aligned with our chemists' domain expertise.

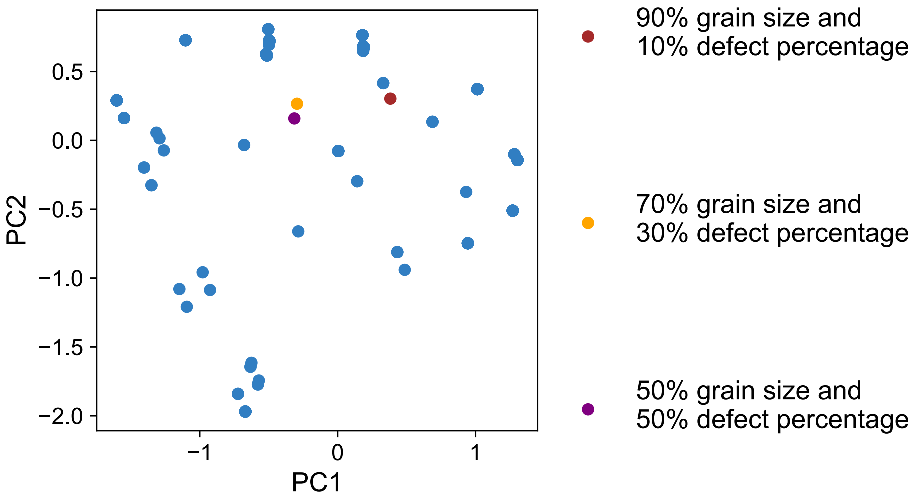

Figure S6: Comparison of the best synthesis conditions identified by the reinforcement learning (RL) models under different reward function weightings. Specifically, we tested three weighting schemes: 90% grain size & 10% defect percentage, 70% & 30% (used in the main manuscript), and 50% & 50%, visualized via principal component analysis (PCA) on the original full dataset containing all three compositions: $AgBiI_4$, Ag_2BiI_5, and Ag_3BiI_6.

Table S1: Best synthesis conditions identified by the reinforcement learning (RL) models under different reward function weightings than the one in the main manuscript.

	**Preheat Temperature (°C)**	**Composition**	**Spin Coating Speed**	**Antisolvent**
**RL with 90:10 grain size to defect weighting**	150	$AgBiI_4$	1000rpm 2s, 6000rpm 30s	None
**RL with 50:50 grain size to defect weighting**	90	$AgBiI_4$	6000rpm 30s	None

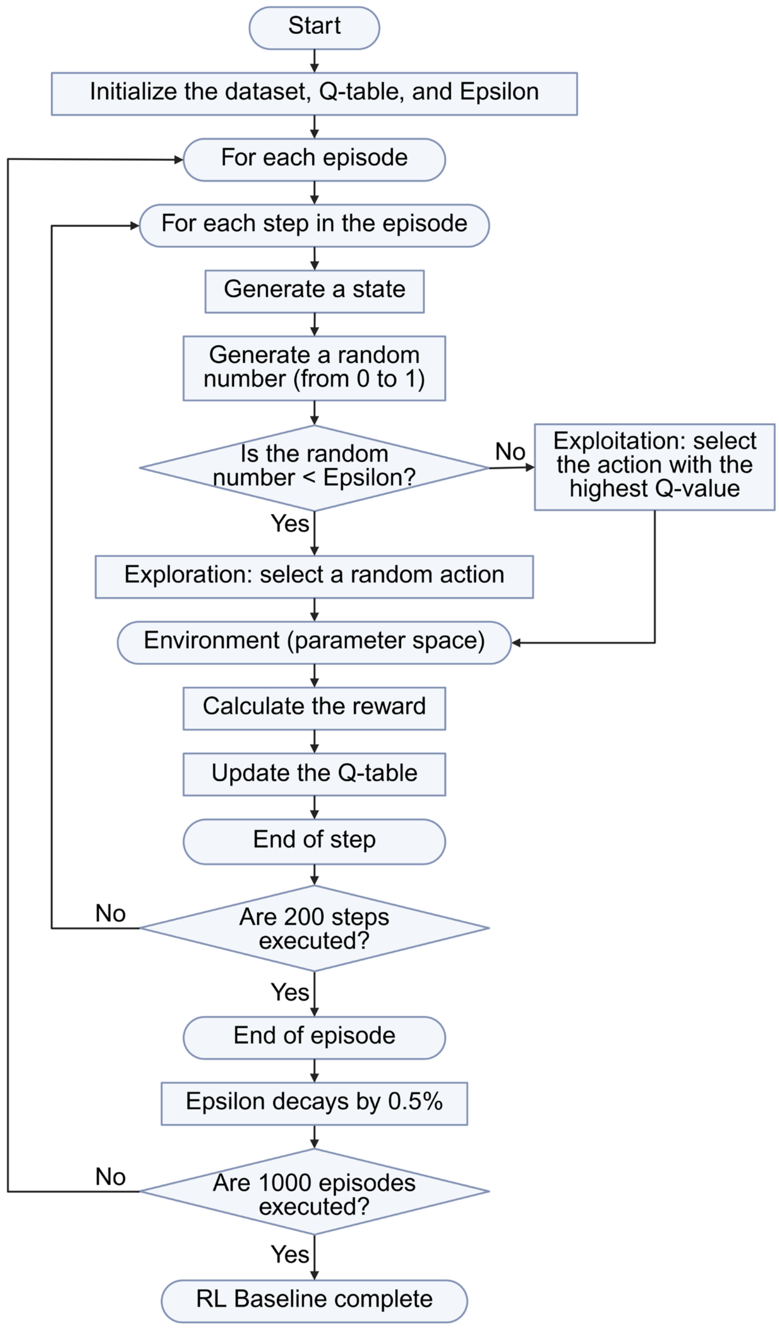

Figure S7: Detailed workflow of the reinforcement learning (RL) baseline approach. This approach performs optimization exclusively within the original dataset by using only the synthesis-condition combinations shown in Figure 4(b).

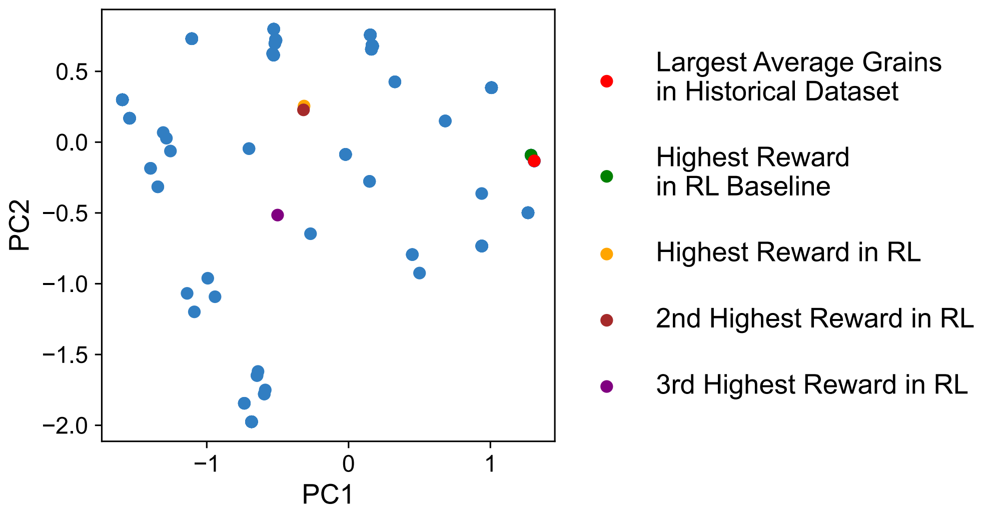

Figure S8: Comparison of the best synthesis conditions identified by the reinforcement learning (RL) baseline and the first, second, and third best synthesis conditions identified by the exploratory RL against the historical dataset's top-performing condition with the largest average grains detected. This plot is visualized via principal component analysis (PCA) on the original full dataset containing all three compositions: $AgBiI_4$, Ag_2BiI_5, and Ag_3BiI_6.

Table S2: Synthesis conditions of the second and third best synthesis conditions identified by reinforcement learning (RL).

	Preheat Temperature (°C)	Composition	Spin Coating Speed	Antisolvent
**The Second Highest Reward in RL**	130	$AgBiI_4$	6000 rpm 30s	None
**The Third Highest Reward in RL**	90	$AgBiI_4$	6000 rpm 30s	None

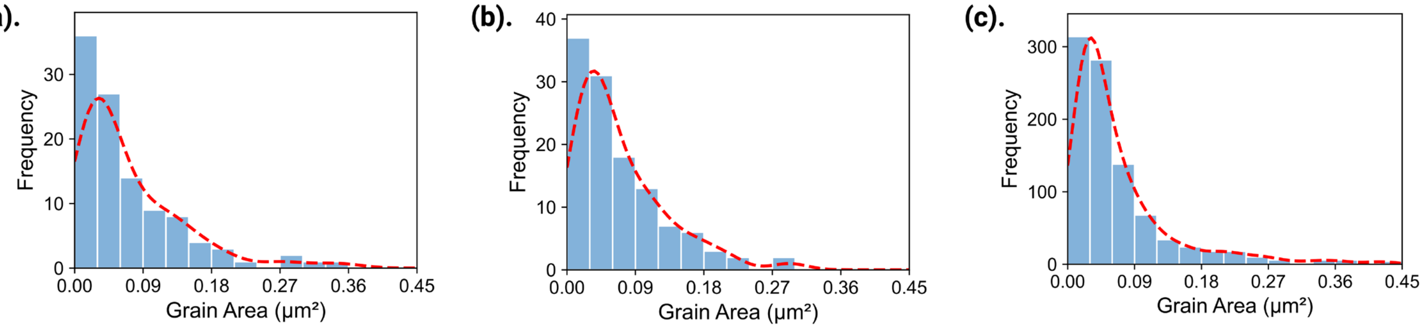

Figure S9: Grain area distributions for the thin films shown in Figure 5(d). (a), The film with the largest average grain size in the historical dataset. (Data acquired from 1 SEM image.) (b), The film achieving the highest reward under the RL baseline model. (Data acquired from 1 SEM image.) (c), The films achieving the highest reward under the exploratory RL model, which includes data from 6 new SEM images outside the training dataset (3 images per sample across 2 independently fabricated films).

Bayesian Optimization (BO)

In addition to employing RL to optimize the synthesis conditions of spin-coated $AgBiI_4$ thin films, we explored BO as an alternative approach. Specifically, we implemented a Gaussian Process (GP)-based BO framework to identify synthesis parameters that maximize grain size while minimizing defect coverage. These metrics were normalized using the same procedure as in the RL method to ensure direct comparability, with grain size assigned a weight of 70% and defect percentage weighted at 30%. The objective function was defined as:

$$f_{objective} = -(0.7 \times (grain\ size)_{normalized} - 0.3 \times (defect\ percentage)_{normalized}) \quad (2)$$

where minimizing $f_{objective}$ corresponds to optimal synthesis conditions. To maintain experimental relevance, the search space was restricted to parameter combinations observed in the dataset.

The BO framework utilized 5 initial samples to build the surrogate model. We then employed four acquisition functions: Expected Improvement (EI), Probability of Improvement (PI), Lower Confidence Bound (LCB), and Thompson Sampling (TS).[5,6] The optimization process iteratively selected parameter sets using these functions, updated the GP model based on observed results, and refined predictions to balance exploration (sampling from uncertain regions) and exploitation (sampling near known optima). While EI, PI, and LCB explicitly maximized acquisition functions, TS sampled directly from the posterior distribution. However, as shown in Figure S10, the optimization did not converge within 20 iterations. This result suggests that GP-based BO may not be well-suited for our dataset, likely due to its limited size, parameter interdependencies, and imbalances.

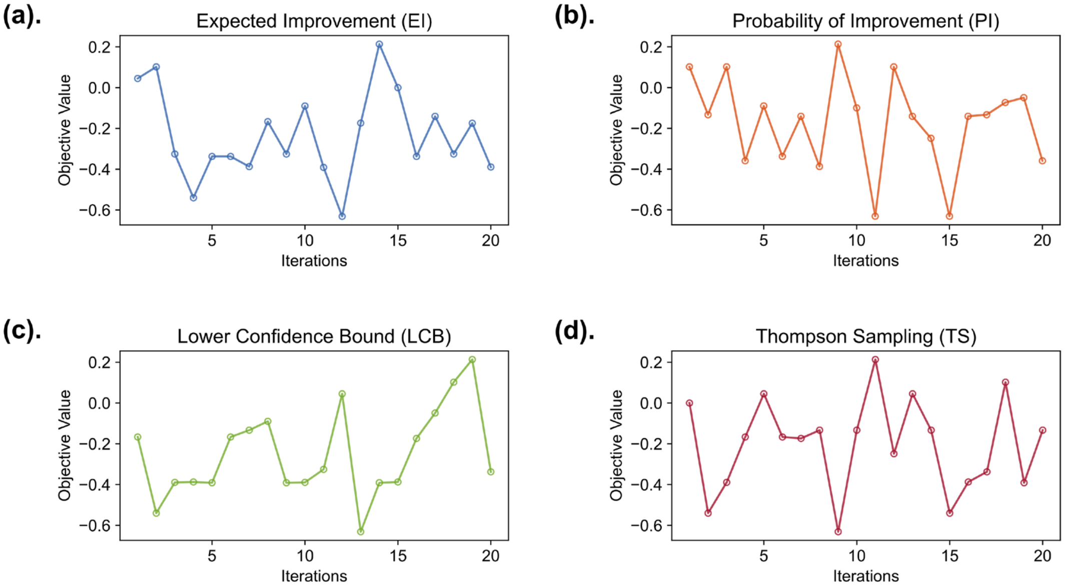

Figure S10: Objective values plotted against the number of iterations for Gaussian Process (GP)-based Bayesian Optimization (BO) using different acquisition functions (shown at the top each subplot). All of them show no clear convergence in 20 iterations.

Random Forest (RF) regression models

Now, to assess the suitability of RF for the optimization task, we first evaluated its predictive performance on our dataset, which is a prerequisite for using it in optimization. Two RF regression models were developed using scikit-learn to predict average grain size and defect percentage based on synthesis parameters.[7] The input features included both categorical variables, such as spin coating speed and antisolvent type, and continuous variables, with categorical features label-encoded for compatibility with the RF algorithm. Hyperparameter tuning was conducted using grid search with 5-fold cross-validation, optimizing parameters such as the number of estimators (n_estimators), the maximum features considered per split (max_features), and the minimum samples required to split a node (min_samples_split). The models were selected based on the lowest root mean squared error (RMSE) across validation folds. While theoretically, RF can accommodate categorical variables and identify critical parameter combinations through feature importance metrics,[8–10] their performance is highly sensitive to data imbalances.[11] The parity plots in Figure S11(a) and S11(b) illustrate that RF struggled to generalize effectively, particularly when trained on unevenly distributed datasets, leading to significant differences between true and predicted values. These results underscore the limitations of RF in capturing complex relationships between synthesis parameters and material properties, further highlighting the challenges of applying traditional machine learning models in this context.

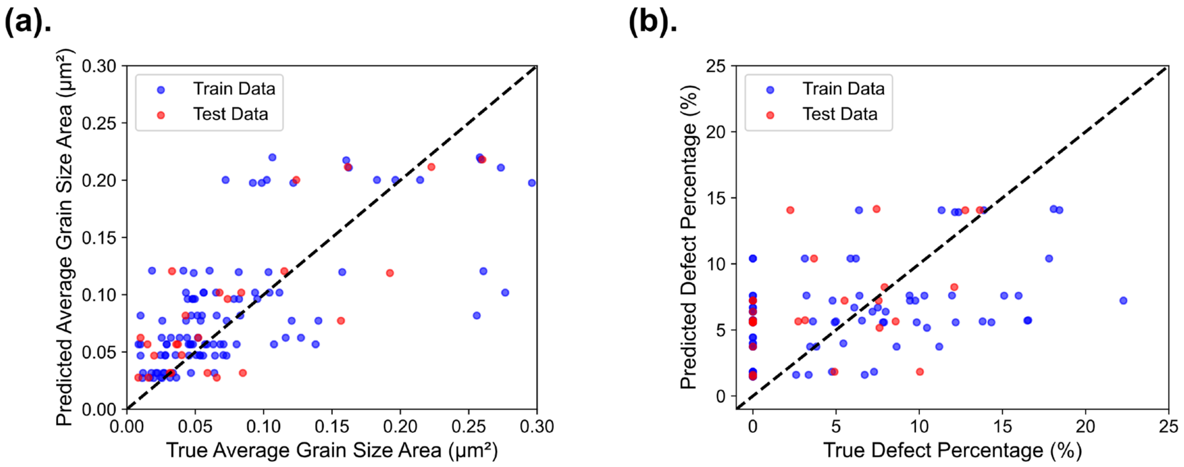

Figure S11: The parity plots illustrating the difference (error) between the true and predicted results. (a), The parity plot of average grain size area. The root mean squared error (RMSE) of the train data and test data are 0.07 μm^2 and 0.06 μm^2, respectively. (b), The parity plot of defect percentage. The RMSE of the train data and the test data are 7.91% and 6.73%, respectively.

References

1. He, K., Zhang, X., Ren, S. & Sun, J. Deep Residual Learning for Image Recognition. in *2016 IEEE Conference on Computer Vision and Pattern Recognition (CVPR)* 770–778 (IEEE, Las Vegas, NV, USA, 2016). doi:10.1109/CVPR.2016.90.
2. Howard, A. G. *et al.* MobileNets: Efficient Convolutional Neural Networks for Mobile Vision Applications. Preprint at https://doi.org/10.48550/ARXIV.1704.04861 (2017).
3. Arzt, M. *et al.* LABKIT: Labeling and Segmentation Toolkit for Big Image Data. *Front. Comput. Sci.* **4**, 777728 (2022).
4. Schneider, C. A., Rasband, W. S. & Eliceiri, K. W. NIH Image to ImageJ: 25 years of image analysis. *Nat Methods* **9**, 671–675 (2012).
5. Gan, W., Ji, Z. & Liang, Y. Acquisition Functions in Bayesian Optimization. in *2021 2nd International Conference on Big Data & Artificial Intelligence & Software Engineering (ICBASE)* 129–135 (IEEE, Zhuhai, China, 2021). doi:10.1109/ICBASE53849.2021.00032.
6. Russo, D. J., Van Roy, B., Kazerouni, A., Osband, I. & Wen, Z. A Tutorial on Thompson Sampling. *FNT in Machine Learning* **11**, 1–96 (2018).
7. Pedregosa, F. *et al.* Scikit-learn: Machine learning in Python. *Journal of Machine Learning Research* **12**, 2825–2830 (2011).
8. De'ath, G. & Fabricius, K. E. Classification and regression trees: a powerful yet simple technique for ecological data analysis. *Ecology* **81**, 3178–3192 (2000).
9. Gomes, C. M. A. & Jelihovschi, E. Presenting the Regression Tree Method and its application in a large-scale educational dataset. *International Journal of Research & Method in Education* **43**, 201–221 (2020).
10. Chen, R.-C., Dewi, C., Huang, S.-W. & Caraka, R. E. Selecting critical features for data classification based on machine learning methods. *J Big Data* **7**, 52 (2020).
11. Breiman, L. Random Forests. *Machine Learning* **45**, 5–32 (2001).
